# Editorial: Microorganisms and microbial technologies for industry and environmental protection

**DOI:** 10.3389/fbioe.2025.1637987

**Published:** 2025-06-26

**Authors:** Jianqiang Lin, Hongbo Hu, Dipesh Dhakal

**Affiliations:** ^1^ State Key Lab of Microbial Technology, Shandong University (Qingdao), Qingdao, China; ^2^ School of Life Sciences and Biotechnology, Shanghai Jiao Tong University, Shanghai, China; ^3^ Department of Medicinal Chemistry, University of Florida, Gainesville, FL, United States

**Keywords:** microbial technology, microbial synthetic biology, industrial mibiology, environmental protection, microbial community

The diverse microbial community in nature, and their metabolic versatility has been utilized as biocatalysis for industrial production, environmental bioremediation and in agriculture. Recently, the cutting-edge advances in the capabilities of the biocatalysts, including microbial cells or their enzymes or microbial consortiums, and developments of innovative technologies for protein engineering for enhanced activity or better physiological properties are crucial for the application of microorganisms or their enzymes in industries. The Research Topic “Microorganisms and Microbial Technologies for Industry and Environmental Protection” has gathered a remarkable Research Topic of five manuscripts that collectively illuminate the transformative potential of microorganisms in addressing global challenges.

## Advancing industrial applications

One of the core themes of this Research Topic is the application of microorganisms in industrial settings. Naeem et al. presented a groundbreaking study on the efficient production of D/L-alanine using recombinant *Escherichia coli* BL21 (DE3). By employing the biobrick approach, they co-expressed three genes (*ald*, *dadX*, and *gdh*) from *Bacillus pseudofirmus* OF4, achieving yields of 6.48 g/L of D-alanine and 7.05 g/L of L-alanine within 3 h under optimized conditions. This work exemplifies the power of metabolic engineering in enhancing microbial productivity and highlights the potential of microbial platforms for large-scale industrial production of essential amino acids.

Ponsetto et al. extended this theme with a comprehensive review of *Clostridial* metabolism for biomass biorefining. These anaerobic bacteria are capable of converting a wide range of substrates, including lignocellulosic waste and C1 gases, into valuable industrial intermediates such as ethanol, butanol, and diols. The authors emphasized the critical role of metabolic engineering in optimizing *Clostridial* strains, with tools such as CRISPR/Cas9 and riboswitches enabling precise gene regulation. These advancements position *Clostridia* as key players in reducing reliance on fossil fuels and mitigating greenhouse gas emissions.

## Addressing environmental challenges

Environmental pollution remains a persistent challenge, particularly the contamination of fossil fuels with sulfur-containing compounds. Bagchi and Srivastava addressed this issue with crucial refinement of microbial biodesulfurization, a promising alternative to conventional hydrodesulfurization methods. Through genetic engineering and enzyme optimization, researchers have enhanced the activity of desulfurizing enzymes (DszC, DszD, DszA, and DszB) involved in the 4S pathway. The 4S pathway (also known as the dsz pathway) is a metabolic route used by certain microorganisms for the biodesulfurization (BDS) of organic sulfur compounds, particularly heterocyclic sulfur compounds like dibenzothiophene (DBT). Sulfur is removed in the form of sulfite (SO_3_^2−^) or sulfate (SO_4_^2−^), depending on the downstream metabolism of the organism, which is the primary product of biodesulfurization. The secondary product of biodesulfurization is the carbon skeleton remains intact as 2-hydroxybiphenyl (2-HBP), which can be further utilized or degraded by the microorganism. This approach not only reduces sulfur emissions but also minimizes the environmental and health risks associated with sulfur pollution.

## Supporting sustainable agriculture

Agriculture faces numerous challenges, including abiotic stressors like salinity and alkalinity. Li et al. investigated the growth-promoting effects of a self-selected microbial community on wheat seedlings in saline-alkali soils. They identified a synergistic blend of *Kocuria dechangensis*, *Rossellomorea aquimaris*, *Bacillus subtilis*, and *Bacillus velezensis* that improved plant physiology and soil health. This microbial agent not only enhanced the biomass and water-use efficiency of wheat seedlings but also regulated enzyme activities, offering a sustainable solution for restoring degraded agricultural lands.

## Accelerating genetic analysis

Efficiency in genetic analysis is crucial for designing critical strategies for advancing microbial technologies. Yuan et al. introduced a novel High-Throughput Genome Releaser (HTGR) designed for rapid and efficient DNA extraction from fungal spores. This device employs a squash-based mechanism to release genomic DNA directly into a 96-well plate, reducing processing time to minutes while maintaining high DNA quality. The compatibility of the HTGR with automated liquid-handling platforms further accelerates strain optimization workflows, making it an invaluable tool for synthetic biology and metabolic engineering projects.

## Broader implications and future directions

Collectively, these contributions demonstrate the multifaceted role of microorganisms in addressing global challenges from synthesis of essential bioproducts to bioremediation of environmental pollution and supporting sustainable agriculture. However, for exploiting their full potential, it requires overcoming several barriers, including scalability, cost-effectiveness, and regulatory approval. Future research should focus on integrating multi-omics approaches, machine learning, and synthetic biology to further optimize microbial performance and expand their applications. For instance, combining microbial technologies with AI-driven predictive modeling could accelerate the discovery of novel enzymes and pathways.

In conclusion, this Research Topic of articles highlights the transformative power of microorganisms and microbial technologies in shaping a sustainable future. As we continue to explore the vast untapped potential of these microscopic organisms, their role in industry and environmental protection will undoubtedly grow even more significant.

